# 

**Published:** 1880-10

**Authors:** 


Vol. I.] TWO DOLLARS A YEAR. SINGLE COPIES 50 CENTS. [No. 4.

### THE ARCHIVES

OF

### COMPARATIVE MEDICINE AND SURGERY.

### A Quarterly Journal

OF THE

### ANATOMY, PATHOLOGY, AND THERAPEUTICS OF ANIMALS.

NEW YORK:

W. L. HYDE & Co., PUBLISHERS, No. 22 UNION SQUARE.

1880.

